# 4-Chloro-2-Isopropyl-5-Methylphenol Exhibits Antimicrobial and Adjuvant Activity against Methicillin-Resistant *Staphylococcus aureus*

**DOI:** 10.4014/jmb.2203.03054

**Published:** 2022-05-06

**Authors:** Byung Chan Kim, Hyerim Kim, Hye Soo Lee, Su Hyun Kim, Do-Hyun Cho, Hee Ju Jung, Shashi Kant Bhatia, Philip S. Yune, Hwang-Soo Joo, Jae-Seok Kim, Wooseong Kim, Yung-Hun Yang

**Affiliations:** 1 Department of Biological Engineering, College of Engineering, Konkuk University, Seoul 05029, Republic of Korea; 2 College of Pharmacy and Graduate School of Pharmaceutical Sciences, Ewha Womans University, Seoul 03760, Republic of Korea; 3 Division of Infectious Diseases, Department of Medicine, Montefiore Medical Center, Albert Einstein College of Medicine, Bronx, NY, USA; 4 Department of Biotechnology, College of Engineering, Duksung Women’s University, Seoul 01369, Republic of Korea; 5 Department of Laboratory Medicine, Kangdong Sacred Heart Hospital, Hallym University College of Medicine, Seoul 07226, Republic of Korea

**Keywords:** MRSA, thymol derivatives, chlorothymol, antimicrobial, synergistic effect, biofilm

## Abstract

Methicillin-resistant *Staphylococcus aureus* (MRSA) causes severe infections and poses a global healthcare challenge. The utilization of novel molecules which confer synergistical effects to existing MRSA-directed antibiotics is one of the well-accepted strategies in lieu of *de novo* development of new antibiotics. Thymol is a key component of the essential oil of plants in the *Thymus* and *Origanum* genera. Despite the absence of antimicrobial potency, thymol is known to inhibit MRSA biofilm formation. However, the anti-MRSA activity of thymol analogs is not well characterized. Here, we assessed the antimicrobial activity of several thymol derivatives and found that 4-chloro-2-isopropyl-5-methylphenol (chlorothymol) has antimicrobial activity against MRSA and in addition it also prevents biofilm formation. Chlorothymol inhibited staphyloxanthin production, slowed MRSA motility, and altered bacterial cell density and size. This compound also showed a synergistic antimicrobial activity with oxacillin against highly resistant *S. aureus* clinical isolates and biofilms associated with these isolates. Our results demonstrate that chlorinated thymol derivatives should be considered as a new lead compound in anti-MRSA therapeutics.

## Introduction

Since the discovery of penicillin, many types of antibiotics have been developed and used [1]. Antibiotics are therapeutic agents that inhibit bacterial growth or kill bacteria. Due to the indiscriminate use of antibiotics, significant selection pressure has been applied to many bacteria [2]. *Staphylococcus aureus* is a pathogen that is widespread in humans and has various virulence factors; it can infiltrate body tissues and cause infection [3]. Although many antibiotics have been used to treat *S. aureus* infections, most of them are ineffective against methicillin-resistant *S. aureus* (MRSA), which first emerged in 1962 and has spread worldwide over time [4]. *mecA*, which is part of the staphylococcal chromosome cassette mec (SCCmec), exists only in MRSA and expresses penicillin-binding protein 2a (PBP2a), which weakens β-lactam sensitivity [5]. Patients infected with MRSA suffer from severe illness and high mortality rates, and their treatment is difficult [6]. Therefore, MRSA has emerged as a global healthcare issue [7]. MRSA can be broadly classified as healthcare-associated MRSA (HA-MRSA), community-associated MRSA (CA-MRSA), and livestock-associated MRSA (LA-MRSA) [8, 9]. Although HA-MRSA was initially noted for its high pathogenicity and mortality rate, CA-MRSA, which is highly infectious and resistant to different antibiotics, has recently emerged as a major problem [10]. The most well-known CA-MRSA strains are LAC (USA300) and MW2 (USA400) [11].

MRSA adapts to the surrounding environment and protects itself by expressing various virulence factors [12]. In particular, unlike other *S. aureus* strains, MRSA protects itself from antibiotics by expressing PBP2a and β-lactamases and forms biofilms that are impermeable to antibiotics [13, 14]. The protection of persister cells by biofilms is a problem in the complete treatment of MRSA [15]. Staphyloxanthin, a golden carotenoid pigment produced by *S. aureus*, is a virulence factor [16]. This pigment not only protects cells from host reactive oxygen species (ROS) and neutrophils but also reduces cell membrane fluidity and prevents the penetration of antimicrobial substances [17, 18]. *S. aureus* does not have movable appendages such as pili or flagella, but it can move through secretion of an amphiphilic substance called phenol-soluble modulin (PSM) [19]. These characteristics can enhance the transmission of MRSA [20]. In addition, PSM itself functions as a virulence factor, causing human cell lysis, inflammatory responses, and biofilm development [21]. Expression of these virulence factors is a great obstacle to patient treatment and helps the bacteria infect hosts [6, 20]. Therefore, inhibition of these virulence factors is important in treating MRSA infections. Compounds such as essential oils have been studied to assess their ability to inhibit these virulence factors [22]. Here, we attempted to identify a novel inhibitory compound that can be used in combination with antibiotics.

Thymol (2-isopropyl-5-methylphenol), a monoterpenoid compound, is a key component of the essential oil of many plants belonging to the *Thymus* and *Origanum* genera [23]. Thymol is widely used for fragrance, food flavoring, and dental treatment [24]. It also has antioxidant, antimicrobial, antitussive, expectorant, antispasmodic, and antibacterial effects [25]. It inhibits a wide range of bacteria, such as *Escherichia coli*, *Salmonella enterica*, and *S. aureus* [26]. In addition, according to a recent study, thymol may have *sarA*-dependent antibiofilm efficacy against MRSA [27].

Carvacrol, a structural isomer of thymol, has antibacterial properties [24]. Carvacrol and thymol exert antibacterial effects through cell membrane disruption, biofilm reduction, inhibition of motility, membrane-bound ATPase, and efflux pumps [24]. Although the effective and diverse antibacterial effects of thymol are well known, little is known about the antibacterial effects of thymol derivatives on MRSA. Therefore, we not only studied the effects of thymol derivatives on CA-MRSA strains LAC and MW2 but also extended the scope to clinical strains to evaluate the antibacterial effect of thymol derivatives against more diverse MRSA strains. Lastly, we analyzed the various phenotypes of the cells that appeared when thymol derivatives were added.

## Materials and Methods

### Strains, Media, Materials, and Culture Conditions

Wild-type (WT) strains of *S. aureus* USA300-0114 (LAC), USA400 (MW2), and their Δagr mutant strains were obtained from Dr. Michael Otto at the Pathogen Molecular Genetics Section, Laboratory of Bacteriology, National Institute of Allergy and Infectious Diseases, U.S. National Institutes of Health. The Δ*sarA* transposon mutant JE2 and the clinical strains were obtained from Jae-Seok Kim, M.D., PhD, Department of Laboratory Medicine, Kangdong Sacred Heart Hospital, Hallym University College of Medicine, Seoul, Korea. Carvacrol (5-isopropyl-2-methylphenol), thymol iodide ([4-(4-iodooxy-2-methyl-5-propan-2-ylphenyl)-5-methyl-2-propan-2-ylphenyl] hypoioditel), 4-isopropyl-3-methylphenol, and chlorothymol (4-chloro-2-isopropyl-5-methylphenol) (Sigma Aldrich, USA) were used as thymol derivatives. The cells were cultured in liquid tryptic soybean broth (TSB). For preculture, a single colony of the strain from a TSB agar plate was inoculated with 5 ml of TSB medium using a sterilized inoculation loop. After that, 1% (v/v) of the cell culture suspension was inoculated into TSB for subsequent cell cultivation at 37°C in a shaking incubator (200 rpm) or a 96-well plate without shaking [28].

### Antimicrobial Susceptibility and Biofilm Formation

To investigate the antimicrobial susceptibility and biofilm formation, 200 μl of culture broth containing serially diluted oxacillin was prepared in a 96-well plate. Precultured cells were inoculated (1% v/v), and the plate was incubated at 37°C for 24 h without shaking. Cell optical density was measured using a 96-well plate reader (Thermo Fisher Scientific, USA). Biofilm formation was analyzed using crystal violet staining [29]. After the supernatant was carefully removed, biofilm fixation was performed with methanol and subsequently air-dried for 24 h. Thereafter, a 0.2% crystal violet solution was added to each well to stain the biofilm for 5 min. The remaining dye was removed and washed twice with distilled water. Finally, absorbance was measured at 595 nm using a 96-well microplate reader (Thermo Fisher Scientific) [28]. As chlorothymol is insoluble in common solvents, including water, ethanol was used as the solvent. Due to the potential toxic effect of ethanol on the cells, the same amount of ethanol without the compound was added to the control, and an inhibitory effec*t* test was performed [30]. Ethanol did not show any inhibitory effect on cell growth even at the volume used with a compound concentration of 512 μg/ml (Fig. S1).

### Motility Assay in a Soft Agar Plate

To determine the change in motility caused by the addition of chlorothymol, we conducted a previously reported soft agar assay [31]. A 20 μl aliquot of precultured cells was centrifuged and resuspended in the same volume of phosphate-buffered saline (PBS). Aliquots (2 μl) of the mixture were dropped onto the center of a 0.24%TSB agar plate and incubated for 10 h at 37°C. All experiments were performed in triplicate [28].

### Staphyloxanthin Extraction and Quantification

Cells were grown in 5 ml of TSB with shaking (200 rpm) for 6, 12, 18, and 24 h at 37°C and harvested by centrifugation (3,000 ×*g*, 20 min). Each sample was then subjected to methanol extraction. The cell pellet was washed once with PBS and centrifuged. After the supernatant was completely removed, the pellet was resuspended in 500 μl methanol and incubated at 55°C for 20 min for staphyloxanthin extraction. Following centrifugation, 200 μl of staphyloxanthin in methanol was obtained. To prevent contamination with the cell pellet, the pigment-containing extracts were filtered through a 0.2 μm syringe filter (Chromdisc, Korea). The amount of pigment in each sample was determined immediately by measuring the optical density at 470 nm using a plate reader spectrometer (Thermo Fisher Scientific) [28].

### Semiquantitative Reverse Transcription Polymerase Chain Reaction (RT-PCR)

Preculture was conducted using 5 ml of TSB, initiated using a single colony from a TSB agar plate, in a shaking incubator at 37°C overnight. Cells were cultured in 5 ml TSB with 1% (v/v) inoculum in a shaking incubator at 37°C for 24 h to extract total RNA. The cells were centrifuged at 3,521 ×*g* for 20 min. Total RNA was extracted using TRIzol reagent, and reverse transcription was performed using Superscript IV Reverse Transcriptase (Invitrogen Co., USA) to generate cDNA, according to the manufacturer’s instructions. Primer Express software v3.0.1 was used to design primers from Thermo Fisher Scientific. Then, 150 bp of amplicons generated from these primers were used to compare gene expression levels. PCR cycle number optimization was performed before semiquantitative PCR to determine the saturated gene expression levels of DNA gyrase subunit B (*gyrB*, the endogenous control) for each template. The optimal cycle number was set to 25 to enable further comparative gene expression analysis. Semiquantitative PCR was conducted using LA Taq with GC buffer I (Takara Bio, Japan) using the methods described in the manual [32].

### Fractional Inhibitory Concentration (FIC) Index Analysis

The FIC index analysis was used to mathematically express the effect of the combination of two other antibacterial agents. The FIC of each antibacterial agent (A and B) was calculated as follows:

$${\text{FIC}}_{\text{A}}={\text{ MIC}}_{\text{A in the presence of B}}/{\text{MIC}}_{\text{A only}}$$

$${\text{FIC}}_{\text{B}}{\text{= MIC}}_{\text{B in the presence of A}}{\text{/MIC}}_{\text{B only}}$$

The FIC index (ΣFIC) is the sum of FIC_A_ and FIC_B_ and is a numerical value of the degree of interaction between the two substances. FIC index of less than 0.5 indicates synergism, 0.5–1 signifies an additive effect, 1–2 means indifference, and higher than 2 indicates antagonism [33,34]. We compared the FIC index values of each well of a 96-well plate and used them to determine the optimal concentration combination of the two antibacterial agents.

### Scanning Electron Microscopy (SEM) Analysis of Cell Morphology

For SEM analysis, after 24 h of cultivation, 1 ml of each sample was collected by centrifugation (3,000 ×*g*, 10 min), washed three times with phosphate buffer (pH 6.8–7.0), and fixed with 2% buffered glutaraldehyde overnight. Glutaraldehyde was added after centrifugation, and the residual glutaraldehyde was removed by washing with phosphate buffer three times. The samples were dehydrated using a gradient of ethanol (v/v) (50%, 70%, 95%, and 100%). Different ratios of ethanol and hexamethyldisilazane (HMDS; 2:1, 1:1, 1:2 v/v) were used for chemical drying; 100% HMDS was used in the final step, and the mixture of HMDS and the samples was mounted on specimen stubs. The HMDS was evaporated overnight in a fume hood. The samples were then coated with gold dust at 5 mA for 300 s, and backscattered electron (BSE) images were acquired using SEM (TM4000Plus; Hitachi, Tokyo, Japan) with an accelerating voltage of 5–15 kV [35].

### MRSA Biofilm Killing

The MRSA biofilm-killing activity of the thymol derivatives was assessed as previously described [36]. *S. aureus* MW2 was grown overnight in TSB broth at 37°C. The overnight culture of cells was diluted 1:200 in TSB supplemented with 0.2% glucose and 3% NaCl. A 13 mm mixed cellulose ester membrane (GSWP01300; EMD Millipore, USA) was placed in each well of a 24-well plate. A 100 μl aliquot of the diluted overnight culture was dispensed into each well and cultured statically in a 37°C incubator. After 24 h, the biofilm-forming membranes were washed three times with 1 ml PBS. Then, 1 ml of PBS containing the desired concentration of antibiotics was added to each well and incubated at 37°C for 24 h. The membranes were then washed three times with 1 ml PBS, placed in 1.5-ml microcentrifuge tubes containing 1 ml PBS, and sonicated in a Bronson ultrasonic bath for 10 min. The samples were serially diluted with PBS and spot-plated on Mueller-Hinton II agar plates. The plate was incubated at 37°C and the colonies were counted to estimate the number of surviving cells.

### Membrane Permeability Assay

The membrane permeability of MRSA was assessed by using the membrane-impermeable DNA-binding dye SYTOX Green (cat. No. S7020; Invitrogen) as previously described [37]. Exponential-phase MRSA MW2 cells were washed three times with PBS and adjusted to an OD_600_ of 0.5. SYTOX Green was added to the washed cells at a final concentration of 5 μM. The samples were incubated for 30 min at room temperature in the dark. A 50 μl aliquot of the sample was added to each well of a black 96-well plate containing the indicated concentrations of the compounds. Fluorescence was measured at room temperature using a multimode plate reader (Cytation 5; BioTek, USA or Infinite M200 Pro microplate reader; Tecan Group Ltd., Mannedorf, Switzerland) at excitation and emission wavelengths of 485 nm and 525 nm, respectively. The Infinite M200 plate reader was equipped at Ewha Drug Development Research Core Center. All experiments were conducted in triplicate.

## Results

### Antibacterial Activity of Thymol Derivatives

**Fig. 1:**
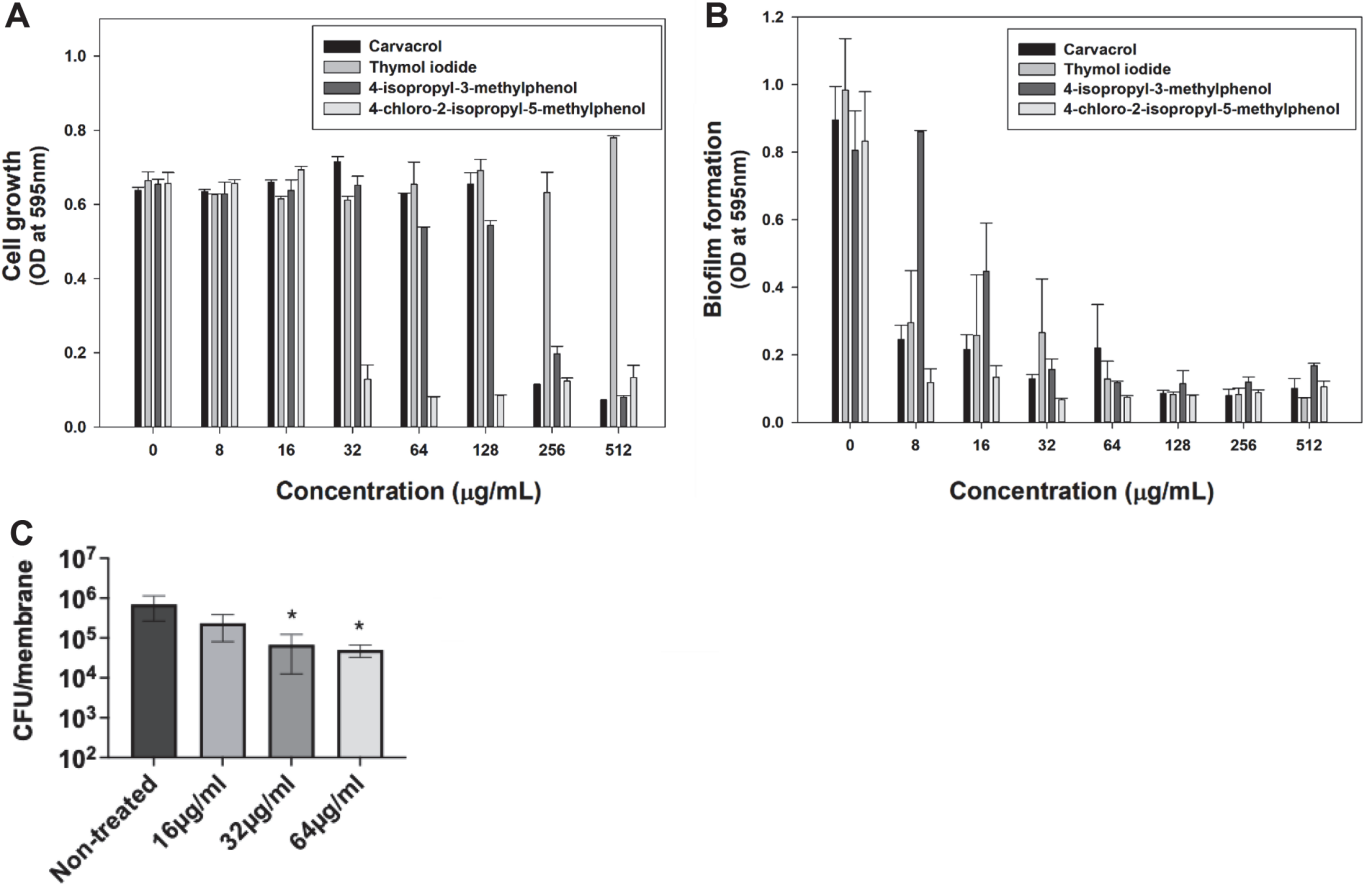
Antibacterial activity and anti-biofilm potency of thymol derivatives against MRSA at various concentrations. (**A**) Growth inhibitory activity against *S. aureus* LAC by various concentrations of thymol derivatives was quantified as a measure of OD_595_. (**B**) Inhibition of *S. aureus* LAC biofilm formation by thymol derivatives was assessed by staining with 0.2% crystal violet. Statistical analysis involved 240 ANOVA (with the level of significance at 5%). (**C**) *S. aureus* MW2 biofilms formed on a 13-mm membrane were treated with the indicated concentrations of chlorothymol for 24 h. The remaining survival cells were enumerated by serial dilution and plating on CaMH II agar plates. The level of detection was 2 × 10^2^ CFU/membrane. Statistical differences between control and treated groups were analyzed by one-way ANOVA and post hoc Tukey test (*p < 0.05).

Although thymol itself has no inhibitory effect on cell growth, we attempted to measure the antibacterial activity of four derivatives. The results suggest that these derivatives have different properties from thymol [27]. To determine their antibacterial activity, their effects on cell growth and biofilm formation were evaluated in the LAC strain. After culturing the cells for 24 h, absorbance was measured at 595 nm. Carvacrol and 4-isopropyl-3-methylphenol showed a minimum inhibitory concentration (MIC) of 512 μg/ml (Fig. 1A). However, thymol iodide showed no growth inhibition effect, even at 512 μg/ml. Chlorothymol had a MIC of 32 μg/ml, showing the most outstanding effect of the four derivatives.

All four derivatives inhibited biofilm formation in a dose-dependent manner. In particular, chlorothymol almost completely inhibited MRSA biofilm formation even at a sub-MIC of 8 μg/ml (Fig. 1B). Next, we assessed the bactericidal activity of chlorothymol on MRSA cells in mature biofilms. As shown in Fig. 1C, chlorothymol at the MIC level of 32 μg/ml led to an approximately 1-log decrease in viability of MRSA biofilm cells. These results indicate that in contrast to thymol, chlorothymol not only inhibits MRSA biofilm formation but also has potency against mature MRSA biofilms. Since chlorothymol exhibited promising antimicrobial activity, we further investigated its effects on MRSA.

### Inhibitory Effect of Chlorothymol on Motility and Staphyloxanthin Production

**Fig. 2:**
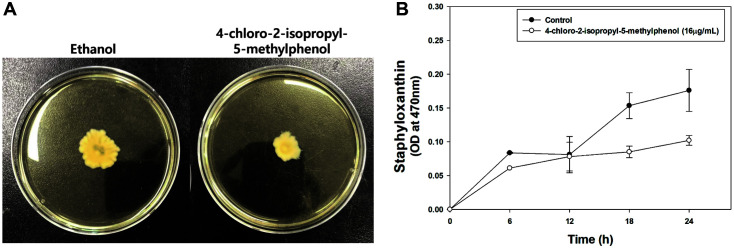
Inhibitory effect of chlorothymol on motility and staphyloxanthin production. (**A**) The experiment was performed in triplicate with similar results. (**B**) Statistical analysis was performed by applying 240 ANOVA with the level of significance at 5%.

Motility characteristics of *S. aureus* play an important role in biofilm formation and host colonization, which are essential for cell survival and adaptation [38]. *S. aureus* was originally considered non-motile; however, it has recently been shown to move through spreading and comet formation under certain conditions, such as on a soft agar plate [19, 31]. It can move through the production of virulence factors called PSMs, amphiphilic α-helical peptides with surfactant properties [38, 39]. We assessed the effect of chlorothymol on the motility of the LAC strains on soft agar plates. When the compound was added, motility significantly decreased compared to the control. The same amount of ethanol was added to the control as to the compound solvent, which did not affect cell growth. (Fig. 2A).

Staphyloxanthin functions as a barrier from the host immune system [16, 17, 41]. It is also known that thymol has staphyloxanthin inhibitory potential against MRSA [42]. Therefore, we evaluated the change in staphyloxanthin production when 16 μg/ml chlorothymol was added. Each sample was harvested at 6-hour intervals to extract staphyloxanthin (the OD_595_ value of each sample was set to 5, and the cell amount was adjusted equally). The decrease in staphyloxanthin production in the sample with chlorothymol showed a tendency to increase over time (Fig. 2B).

### Effect of Chlorothymol on Major Virulence-Related Genes

**Fig. 3:**
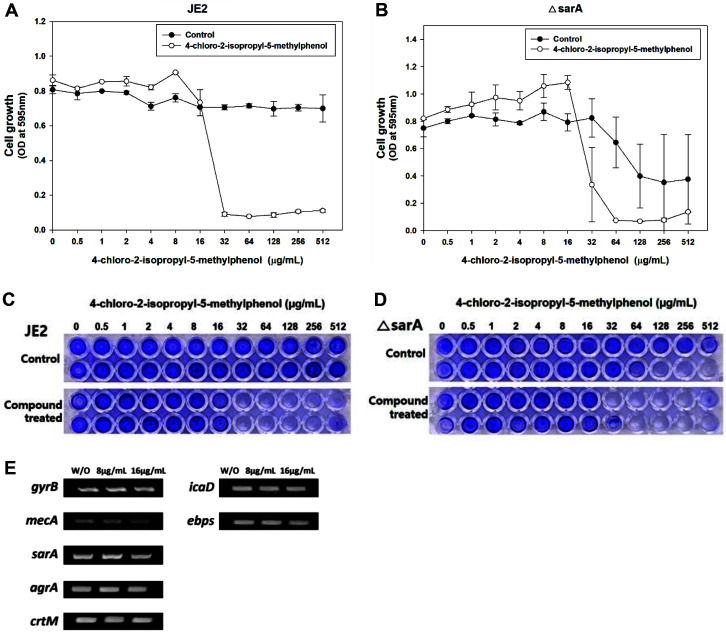
Effect of chlorothymol on Δ*sarA* mutant and semiquantitative RT-PCR of different virulence factorrelated genes. (**A, B**) Statistical analysis was performed by applying 240 ANOVA with the level of significance at 5%. (**C, D**) Images below show post crystal violet staining in 96-well plates cultivated at 37°C for 24 h. (**E**) Semi-quantitative PCR of *mecA*, *sarA*, *agrA*, *crtM*, *icaD*, and *ebps* genes. *gyrB* is used as an endogenous control.

Thymol and its analog carvacrol alter the expression of several *S. aureus* virulence factors including the global regulator *sarA* and the accessory gene regulator *agr* [27, 43, 44]. Thus, we sought to determine whether chlorothymol also modulates *S. aureus* virulence factors. Since thymol inhibits *S. aureus* biofilm formation in a *sarA*-dependent way [27] and *sarA* regulates *agr* [45], we assessed the role of *sarA* and *agr* on the antimicrobial activity of chlorothymol using *sarA* and *agr* deletion mutants, respectively. The MIC of chlorothymol was 32 μg/ml against both the parental control strain JE2 and its *sarA* deletion mutant (Figs. 3A and 3B). This compound completely inhibited the biofilm formation of both MRSA JE2 and the *sarA* mutant at 32 μg/ml (Figs. 3C and 3D). Consistently, the MIC of chlorothymol was 32 μg/ml against both the parental control strain LAC and its *agr* deletion mutant; its biofilm inhibition concentration was approximately 16 μg/ml (data not shown). These results indicate that unlike thymol, chlorothymol inhibits MRSA growth and biofilm formation in either *sarA*- or *agr*-independent manners.

Next, we evaluated the expression levels of virulence factor genes, such as *sarA*, *agrA*, the antibiotic resistance gene *mecA* [46], the diapophytoene synthase gene *crtM*, the intercellular adhesion gene *icaD*, and elastin-binding protein gene *ebpS* [47, 48] after treating *S. aureus* with chlorothymol at 8 and 16 μg/ml. There was no significant difference in the expression levels of these genes (Fig. 3E), indicating that unlike thymol and carvacrol, chlorothymol does not target these virulence-related genes.

### Synergetic Effect of Chlorothymol with Oxacillin

As the antibacterial effect of thymol derivatives was confirmed, the synergetic effect of thymol derivatives with other antibiotics was evaluated. Cell growth inhibition was observed by adding 8 μg/ml oxacillin and various concentrations of thymol derivatives to the LAC strain. Carvacrol showed a MIC of 64 μg/ml (Fig. S3A). We found a synergetic effect compared to 256 μg/ml when carvacrol was used alone. In the case of thymol iodide, when used alone, even at a concentration of 512 μg/ml, there was no significant inhibitory effect on LAC, and the combination with oxacillin showed no synergistic effects (Fig. S3B). In the case of 4-isopropyl-3-methylphenol, the MIC was reduced to 128 μg/ml when combined with oxacillin, compared to the MIC of 512 μg/ml alone (Fig. S3C). In the case of chlorothymol, the inhibitory effect was strong enough to show MIC, even at a concentration of 8 μg/ml (Fig. S3D). We found a significant synergetic effect with oxacillin compared to 32 μg/ml when chlorothymol was used alone.

**Fig. 4:**
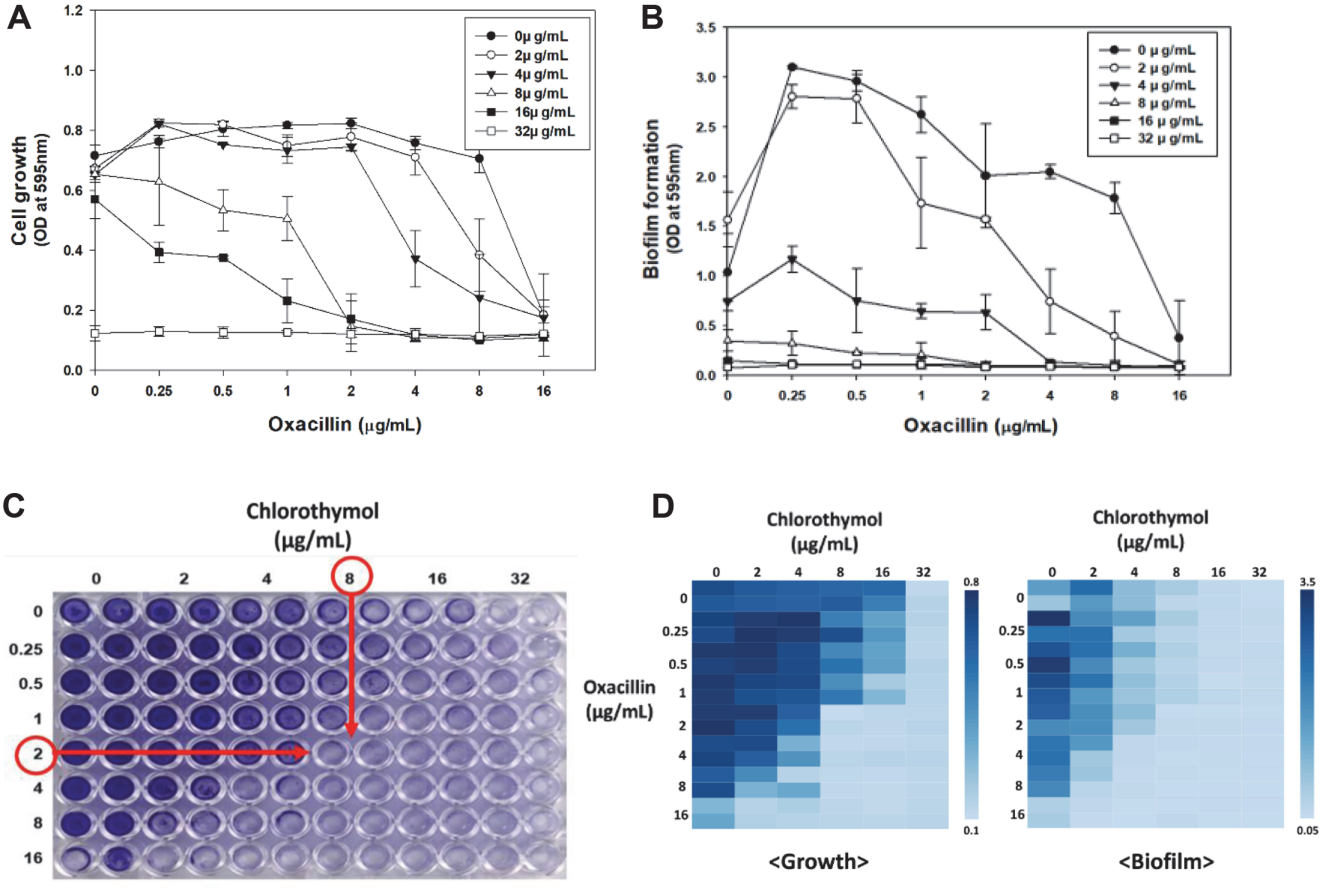
Synergetic effects between oxacillin and chlorothymol. (**A, B**) Statistical analysis was performed by applying 240 ANOVA with the level of significance at 5%. (**C**) Images below show post crystal violet staining in 96-well plates cultivated at 37°C for 24 h. (**D**) The results of the checkerboard assay for confirming the synergetic effect of oxacillin and chlorothymol were presented as heatmaps. Each legend represents the absorbance value at OD_595_.

**Fig. 5:**
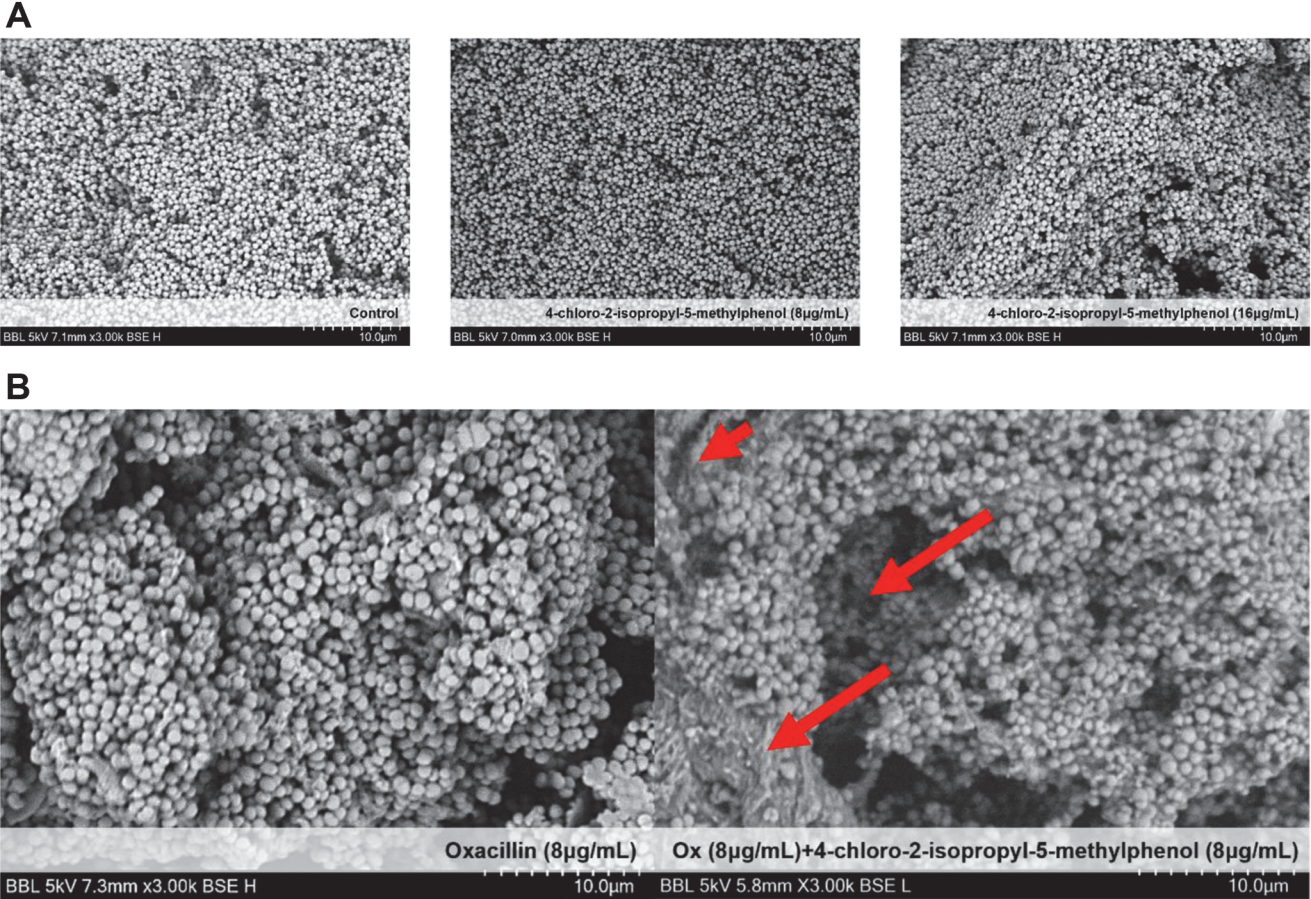
Effects of cell size, density, and synergetic effect with oxacillin when chlorothymol was added, as evaluated using SEM. (**A**) Control and LAC with 8 μg/ml and 16 μg/ml of chlorothymol were incubated in a shaking incubator at 37°C for 24 h. (**B**) Control and LAC, to which 8 μg/ml of oxacillin and 8 μg/ml of chlorothymol were added, were incubated in a shaking incubator at 37°C for 24 h.

Therefore, we conducted a checkerboard assay to determine the optimal concentration for the synergetic effects of oxacillin and chlorothymol. Using the checkerboard assay, we calculated the FIC index values. The lowest FIC index value of cell growth was approximately 0.3125 (each concentration was oxacillin 2 μg/ml, chlorothymol 8 μg/ml), which was less than 0.5. This confirmed a synergetic effect between oxacillin and chlorothymol (Figs. 4A and 4D). When 2 μg/ml oxacillin and 8 μg/ml chlorothymol were used together, we found that inhibition of growth and biofilm was greatly increased. (Figs. 4B-4D). We found that a significant MIC reduction effect could be obtained with the minimal use of antibiotics. To check how the surface of the cell changes with chlorothymol, the cell size and density were observed using a SEM. When chlorothymol was added at concentrations of 8 and 16 μg/ml, there was a decrease in cell density and growth (Fig. 5A). However, there was no significant change in cell size up to 16 μg/ml chlorothymol.

The synergistic effects of oxacillin and chlorothymol were confirmed by SEM. When only oxacillin was used, the cell size was mostly uniformly distributed, but when treated with 8 μg/ml chlorothymol, not only the density of cells but also the size of many cells decreased (Fig. 5B). As a result, we found through SEM that chlorothymol had its own antibacterial effect as well as a synergetic effect with oxacillin.

### Effect of Chlorothymol on Clinical Strains-Synergetic Effect with Oxacillin

The antimicrobial activity of chlorothymol on several clinical strains isolated from patients was also evaluated. Most clinical strains showed strong resistance even at high concentrations of oxacillin (Table 1). Ethanol alone was used on control cells and did not significantly affect cell growth or biofilm formation in the clinical strains, indicating there was no impact of ethanol use on the chlorothymol results (Figs. S2A and S2B). When chlorothymol was added, cell growth was significantly inhibited in most clinical strains at 32–64 μg/ml (Table 2); only MRSA 12779 was resistant. A biofilm inhibition test was also performed on clinical strains. When chlorothymol was added, biofilm formation by all clinical strains, including MRSA 12779, was inhibited to below 128 μg/ml (Table 3).

Additionally, we evaluated the synergetic effect of chlorothymol and oxacillin in clinical strains. In previous studies, 16 μg/ml chlorothymol showed little cell growth inhibition in clinical strains. However, by combining the same concentration of chlorothymol and various concentrations of oxacillin, we succeeded in inhibiting the growth of all strains (Table 2). In addition, although MRSA strain 12779 was resistant to chlorothymol, we were able to inhibit its growth by the combination of 16 μg/ml chlorothymol with 256 μg/ml oxacillin (Table 2). Consistently, chlorothymol acted synergistically with oxacillin against the biofilm formation of these clinical MRSA strains (Table 3).

### Chlorothymol Disrupts Membrane Integrity of MRSA

**Fig. 6:**
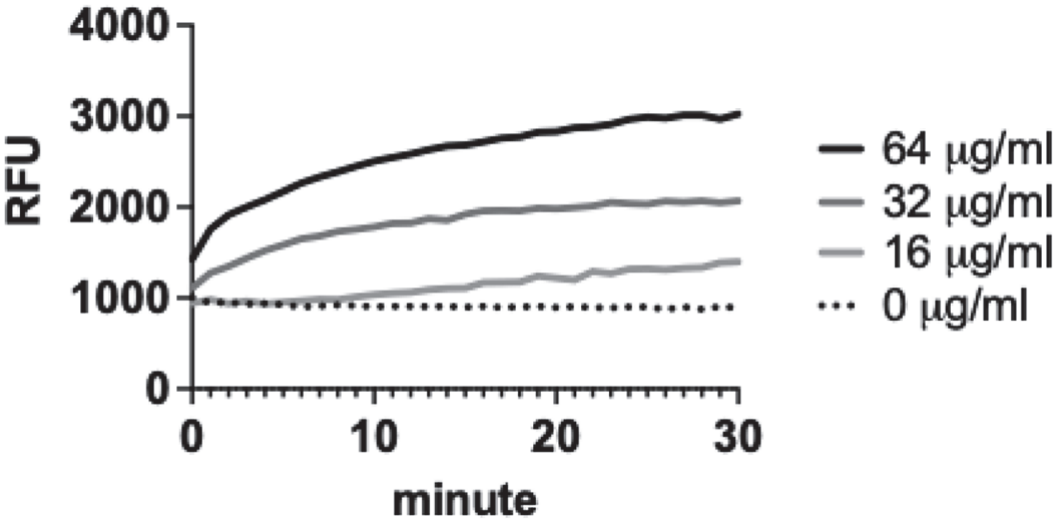
Chlorothymol induces rapid membrane permeabilization of *S. aureus* MW2. After treating MRSA MW2 cells with the indicated concentrations of chlorothymol, the uptake of SYTOX Green was measured by detecting fluorescence intensity (Ex 485 nm, Em 525 nm). The graph represents three independent experiments. Error bars are not shown for clarity.

Membrane-active antimicrobials often exhibit bactericidal activity against MRSA biofilms and synergism with other antibiotics [37, 49]. Thus, we hypothesized that chlorothymol targets the bacterial membrane. To test this, we treated MRSA MW2 cells with a range of concentrations of chlorothymol and measured the cellular uptake of the membrane-impermeable DNA-binding fluorescent dye SYTOX Green. As shown Fig. 6, chlorothymol induced rapid membrane permeabilization of MRSA MW2 cells. This result indicates that the antimicrobial activity of chlorothymol results from disruption of membrane integrity.

## Discussion

In this study, we assessed the antibacterial effects of four thymol derivatives against MRSA and identified that chlorothymol had the most potent effects. We found that chlorothymol not only inhibited MRSA growth, it also prevented MRSA biofilm formation, killed MRSA biofilm cells, decreased MRSA motility and the production of staphyloxanthin, and functioned synergistically with oxacillin. Additionally, chlorothymol alone or in combination with oxacillin was effective in most clinical strains.

Thymol inhibited the expression of *sarA* in MRSA and inhibited the expression of other *sarA*-regulated genes [27]. As a result, the expression of *ica* genes was inhibited and biofilm formation was suppressed [27]. However, our semiquantitative RT-PCR results revealed that chlorothymol did not affect the expression of *icaD* or *sarA*. Further unlike thymol, chlorothymol did not change the expression levels of virulence genes. Therefore, we concluded that the anti-biofilm potency of chlorothymol contributes to its antimicrobial activity.

The antimicrobial synergism between chlorothymol and oxacillin most likely results from the chlorothymol-induced membrane disruption and subsequent promotion of cellular uptake of oxacillin. Indeed, the disruption of the bacterial cytoplasmic membrane is known to increase the permeability of oxacillin[49]. Chlorothymol with hydrophobic properties has a strong affinity with the membrane lipid bilayer, which may facilitate its embedment into the lipid bilayers and eventually cause the reduction of permeability barrier function [50]. Thus, it is highly possible that the concentration of oxacillin increases in chlorothymol-treated MRSA cells.

This study determined the thymol derivative chlorothymol to have the best antibacterial effect among the four thymol derivatives. Chlorothymol significantly inhibited the growth and virulence factors of MRSA. In addition, it showed a synergistic effect with oxacillin and inhibited the growth of all clinical strains. Considering the antimicrobial and adjuvant properties of chlorothymol, chlorothymol and other thymol derivatives have potential to be developed as new therapeutics against deadly MRSA infections.

## Supplemental Materials

Supplementary data for this paper are available on-line only at http://jmb.or.kr.

## Figures and Tables

**Table 1:** Clinically isolated strain characteristics and oxacillin MIC.

Name	Type	SCCmec Type	Oxacillin MIC (μg/ml)	Spa Type	MLST (ST)
2065	MRSA	III	1024	t037	239
6230	MRSA	IV	128	t324	72
6288	MRSA	III	1024	t037	239
7557	MRSA	II	1024	t9353	5
7875	MRSA	IV	128	t664	72
8471	MRSA	II	1024	t9353	5
9291	MRSA	II	1024	t601	5
12779	MRSA	II	1024	t2460	5
14278	MRSA	II	1024	t9353	5
14459	MRSA	IV	1024	t324	72
28984	MSSA	-	1	-	30
28985	MRSA	IV	64	-	30

**Table 2:** MIC of chlorothymol and 16 μg/ml of chlorothymol and oxacillin for clinically isolated strains.

Name	Type Ch	lorothymol MIC (μg/ml)	Oxacillin MIC (μg/ml)	16 μg/ml Chlorothymol with Oxacillin MIC (μg/ml)
2065	MRSA	64	1024	64
6230	MRSA	32	128	16
6288	MRSA	64	1024	32
7557	MRSA	64	1024	512
7875	MRSA	64	128	0.5
8471	MRSA	64	1024	512
9291	MRSA	64	1024	512
12779	MRSA	-	1024	256
14278	MRSA	64	1024	512
14459	MRSA	64	1024	0.5
28984	MSSA	64	1	0.5
28985	MRSA	64	64	0.5

**Table 3:** BIC of chlorothymol and 16 μg/ml of chlorothymol and oxacillin for clinically isolated strains.

Name	Type Ch	lorothymol BIC (μg/ml)	Oxacillin BIC (μg/ml)	16 μg/ml Chlorothymol with Oxacillin BIC (μg/ml)
2065	MRSA	128	1024	0.5
6230	MRSA	64	128	16
6288	MRSA	64	1024	8
7557	MRSA	16	1024	0.5
7875	MRSA	64	512	512
8471	MRSA	64	1024	256
9291	MRSA	32	1024	128
12779	MRSA	32	512	64
14278	MRSA	32	1024	256
14459	MRSA	32	512	512
28984	MSSA	32	0.5	0.5
28985	MRSA	16	512	128

The biofilm inhibition concentration (BIC) was set to an OD_595_ value less than 0.3. Statistical analysis was performed by applying 240 ANOVA with a level of significance of 5%.
