# Supplementary material for: 4-Chloro-2-Isopropyl-5-Methylphenol Exhibits Antimicrobial and Adjuvant Activity against Methicillin-Resistant Staphylococcus aureus
Source: J Microbiol Biotechnol. 2022 May 6;32(6):730–9. doi: 10.4014/jmb.2203.03054 (PMC9628901; doi:10.4014/jmb.2203.03054)
Supplement: Supplementary file 1 [file jmb-32-6-730-supple.pdf]

J. Microbiol. Biotechnol.
https://doi.org/10.4014/jmb.2203.03054

(A)

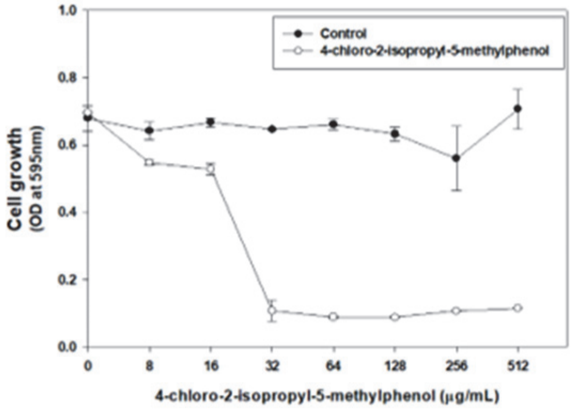

(B)

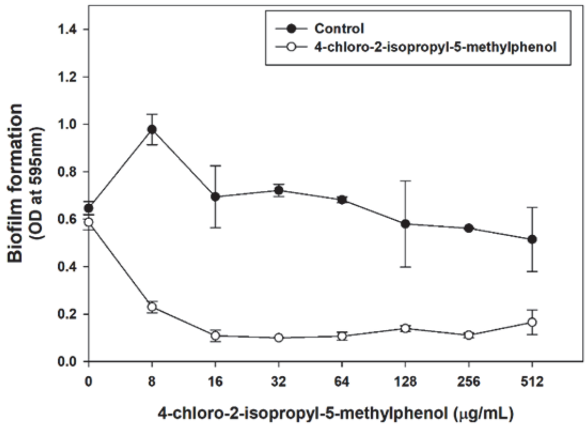

(C)

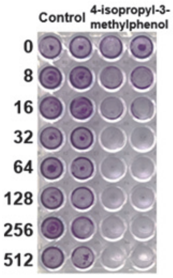

Figure S1. Comparing the effect of ethanol on cell growth and biofilm formation of LAC with the effect of chlorothymol.

(A, B) The same volume of ethanol as the ethanol contained in each concentration of chlorothymol used was added to the control. Statistical analysis was performed by applying 240 ANOVA with the level of significance at 5%. (C) Images below exhibit after crystal violet staining in 96-well plates, which were cultivated at 37℃ for 24 h.

(A)

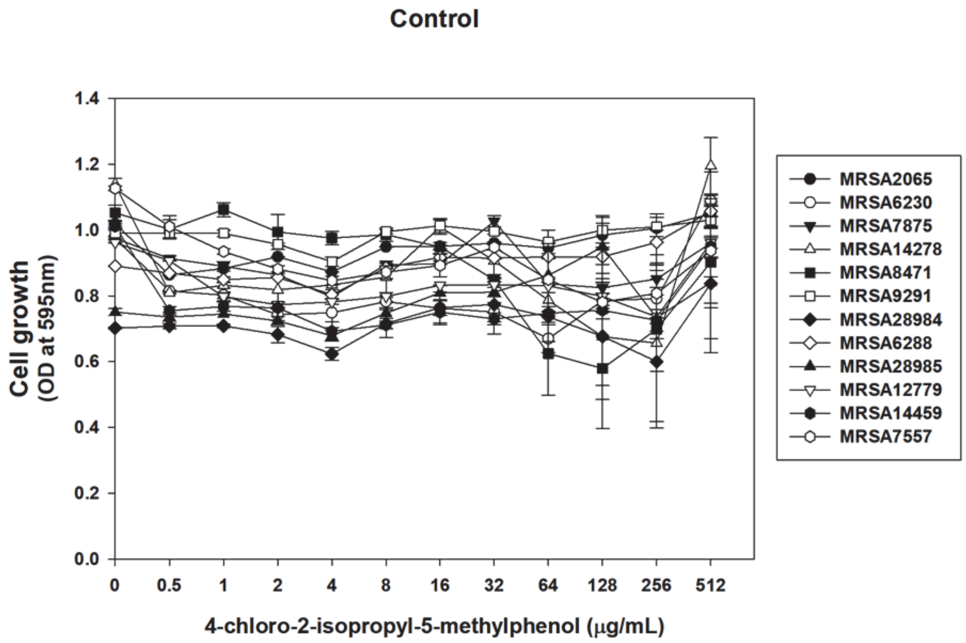

(B)

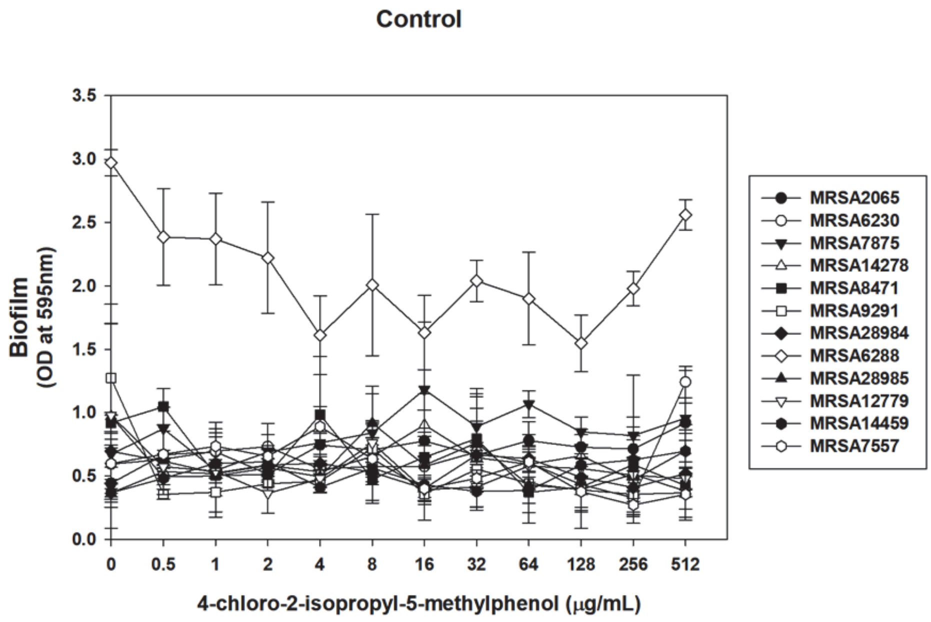

Figure S2. Effect of ethanol on cell growth and biofilm formation of different clinical strains.

(A, B) The same volume of ethanol as the ethanol contained in each concentration of chlorothymol used was added to the control. Statistical analysis was performed by applying 240 ANOVA with the level of significance at 5%.

(A)

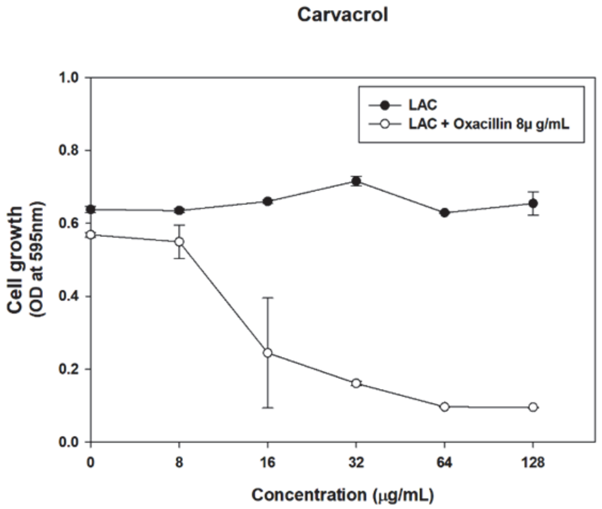
Carvacrol
LAC
LAC + Oxacillin 8μ g/mL
Cell growth (OD at 595nm)
Concentration (μg/mL)

(B)

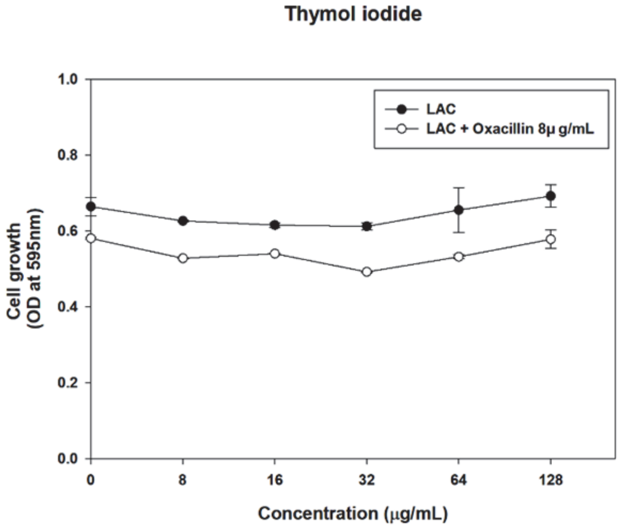
Thymol iodide
LAC
LAC + Oxacillin 8μ g/mL
Cell growth (OD at 595nm)
Concentration (μg/mL)

(C)

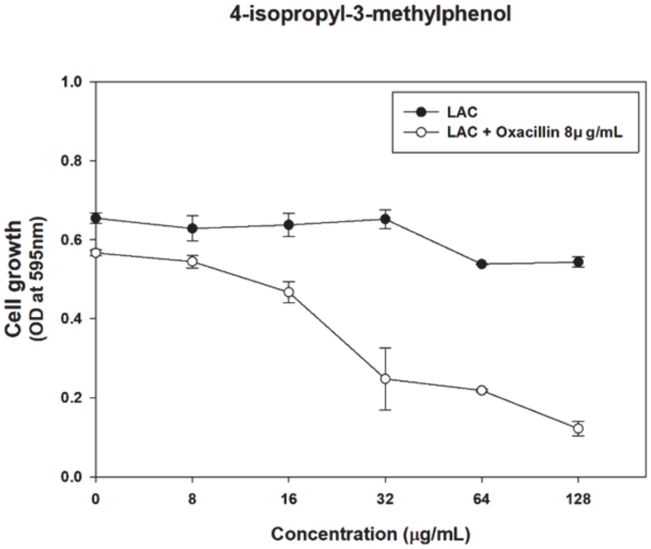
4-isopropyl-3-methylphenol
LAC
LAC + Oxacillin 8μ g/mL
Cell growth (OD at 595nm)
Concentration (μg/mL)

(D)

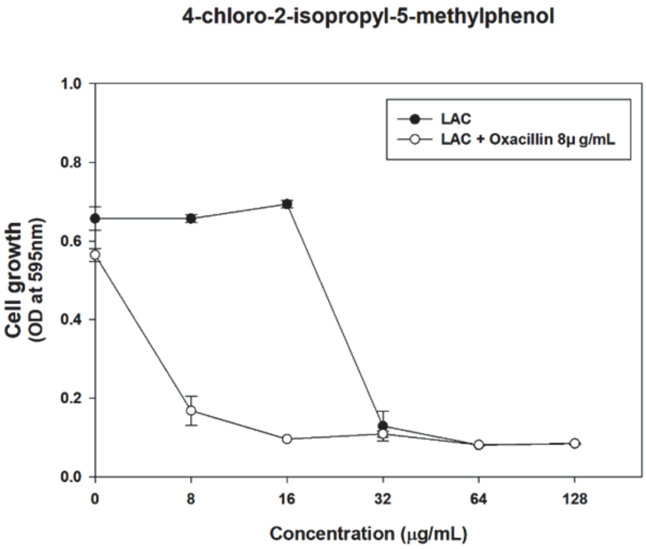

Figure S3. Synergetic effects between oxacillin and several thymol derivatives.

(A, B, C, D) Statistical analysis was performed by applying 240 ANOVA with the level of significance at 5%.
